# Notes of the Week

**Published:** 1921-07-30

**Authors:** 


### NOTES OF THE WEEK.

### A LORD LISTER MEMORIAL.

Last week was presented to the Royal College of Surgeons of Edinburgh a portrait of the late Lord Lister, being a full-sized copy of the portrait by W. Ouless, R.A., now in the possession of the English College. The gift comes from the President, Dr. George Mackay. Lord Lister entered upon his great surgical career as a house surgeon to Professor Syme in the old Royal Infirmary at Edinburgh, and was later a lecturer in surgery at the Extra-Mural School and an assistant surgeon to the Infirmary. Subsequently he succeeded Syme in the Chair of Clinical Surgery in the University.

### THE LATE DR. HAULTAIN.

The Edinburgh Medical School has sustained a severe loss by the death of Dr. F. W. N. Haultain, on July 20. After graduating in 1883 at Edinburgh, he proceeded to the Universities of Prague and Vienna specially to study obstetrics and gynæcology. He was gynæcologist to the Church of Scotland Deaconess' Hospital, and instrumental in starting the Hospital for Women in Archibald Place. He served the full term of office as a physician to the Royal Maternity Hospital, where the excellence of his teaching became widely known. For some time he was assistant gynæcologist to the Royal Infirmary, and four years ago he was elected by the Royal College of Physicians to the Board of Management of that institution. He leaves a widow and one son, who is at present assistant to the Professor of Midwifery in Aberdeen.

### A SUCCESS FOR ECONOMY AT CARDIFF.

Very gratifying figures are recorded at Cardiff, which show that the Finance Committee of King Edward VII. Hospital, and, indeed, other committees also, have done much to reduce expenses and to improve economy. At the monthly meeting, held last week, Mr. Jabez Jones, Chairman of the Finance Committee, reported that the expenditure for the month of June was little more than half what it had been in June 1920—namely, £4,736, instead of £8,362. The principal savings are about £800 on the main hospital and £2,650 on the new extensions account, while the maternity hospital and Lanesworth House have also been run more cheaply. These figures can hardly be too widely known, for allegations have sometimes been made of unnecessary expense, though these suggestions have always been challenged. It seems well now to add that the net debt is about £45,000 and the weekly deficit about £500. But efforts at economy, however successful, can never suffice to finance a hospital. What is needed now is an effort among the local public of South Wales equal in generosity to that already made toward economy by the committees of King Edward VII. Hospital.

### IRISH HOSPITALS AND MEDICAL SECRECY.

The Dublin newspapers greet with approval the attitude of the members of the British Medical Association in regard to the sacredness of the doctor's obligation to respect the confidences of his patients. It is hoped that the attitude of the members may become the official policy of the British Medical Association, and this attitude encourages the Irish doctors, whose opposition made the recent Dublin Hospitals Order a dead-letter, to persist, though, we understand, the order has never been withdrawn. It is remarked the recent feeling shown at the discussions at the British Medical Association makes the withdrawal of the order "the best policy for all concerned." A reason more likely perhaps to be welcome to the officials is provided by the truce in Ireland, since the order seems to have been inspired by the military events now happily suspended.

### A WEEK'S HOLIDAY FOR FIVE SHILLINGS.

Apart from the danger of making a certain number of enemies by sending, unasked, books of tickets, involving sometimes the trouble of returning them or possible leakage by their loss, the London Hospital Carnival scheme has in it the elements of success. It is claimed that a week's enjoyment may be obtained for five shillings, as each book of tickets contains vouchers covering all the days in Carnival week at Stamford Bridge between July 25 and 30. He would be a glutton for pleasure, though, who could stand six days of the galaxy of attractions that are promised. It is hoped that a substantial sum will be raised towards the fund for reopening the closed 200 beds at the London.

### THE VALUE OF ASYLUM VISITORS.

An interesting matter has been brought to light in connection with Menston Asylum and the Wakefield Board of Guardians. Members of a deputation which recently visited the asylum reported that a certain patient was quite cured. The clerk, therefore, was instructed to write to the medical superintendent, who replied that the man was only greatly improved, and that his future would depend upon the conditions in which his discharge would place him. Discussing this reply at a meeting of the board, a guardian said that the matter seemed strange, because twelve months ago a similar report was received from a previous deputation. The Chairman explained that the case was one of considerable improvement only. But he was glad that the matter had been made public, because it would be readily admitted that it was essential to send deputations to visit the patients regularly. It was decided to renew inquiries in three months' time. Deputations indeed are useful not only to the patients and to the bodies from which they come, but to the public, and are the best means of dispelling rumours like that which alleges that patients are kept in asylums after their recovery because they are found "useful."

### PAYING PATIENTS IN POOR-LAW HOSPITALS.

Although it has never been definitely settled whether it is not *ultra vires* for Poor-law Guardians to receive paying patients in their establishments, the Manchester Board of Guardians have conferred with the representatives of the medical profession in Manchester as to the conditions under which such an arrangement, if allowable, can be carried out. Under the scheme agreed on the administrative superintendence of the hospitals would be entirely in the charge of the medical superintendents, but in respect of beds available for cases sent by a medical practitioner the latter will be allowed, if he desires, to attend, treat, and operate himself, with the assistance of the medical and nursing staff of the hospital; but the medical superintendent would have the final voice in the question of the admission of the patients. The Guardians suggest that any tariff of fees for attendance and treatment by the practitioner should be arranged by the Medical Association, but he may, if he desires, attend and treat gratuitously any patient recommended by him who could only afford the standard hospital fees. With regard to the privileges of sending and treating patients, visiting staffs of hospitals shall have the same privileges as a general practitioner. These rules are of considerable interest, as the same question might be under discussion elsewhere. Other areas might do worse than to get similar regulations embodied in any schemes which are drawn up by their Boards of Guardians.

### VOLUNTARY HOSPITALS IN SUSSEX.

The honorary acting staffs of the voluntary hospitals in Sussex, being convened by the British Medical Association, met recently to consider various points arising out of the financial position of the hospitals of the district, and they had before them the recommendations of the staffs of the English and Welsh hospitals, who met in London last December, which they endorsed with modifications and additions. With regard to a State subsidy, it was agreed that to meet the present necessities the State should make a temporary grant through some central hospital fund in a form which is proportional to the amount received from voluntary and other contributions. With regard to the recommendation that part of patients' payments should be allocated to a fund under the control of the honorary medical staff, it was decided that this arrangement should be brought into force only in the event of payments being made for maintenance by some contributory method or State aid or rate aid, but not in cases of payments made by individuals. The above recommendations were to be sent to the Minister of Health and to the Chairman of the December Conference above alluded to.

### ANOTHER HOSPITAL EXPERIMENT.

With a deficit of only £111 on the past year, the Providence Free Hospital, St. Helens, Lancashire, is in a relatively happy position. But it also claims attention because it has lately started a special scheme to provide medical and surgical assistance for weekly subscribers where such is advised by the medical attendant. This, in the absence of precise details, appears to be a local form of the so-called insurance plan advocated by the Voluntary Hospital Commission in its recent report, and now being tried on the Sussex or Oxford plan by certain hospitals in London. It is noteworthy that the Providence Free Hospital already receives over £3,500 a year from working-men's subscriptions, and the new plan, it is hoped, will induce the works which do not at present subscribe to join those already contributing. The nursing is performed by the sisters of the Institute of the Poor Servants of the Mother of God who have been again thanked for their devoted services.

### ST. THOMAS'S AND THE FINANCIAL STRINGENCY.

As the Treasurer of St. Thomas's Hospital emphasised at the hospital's recent prize distribution the system of patients' payments has not been adopted at that institution, where efforts are directed to securing from patients on their departure a free gift according to their means. Added to this, St. Thomas's is making a special effort, we are glad to learn, to enlist the sympathies and assistance of the staff and employers in business houses and factories. The response so far has been encouraging, and in many cases the amounts subscribed by employees are duplicated by the employers. The Gas Light and Coke Company have also made a welcome concession by a special discount of 5 per cent., which is said to be equivalent to a monetary gift of £200 to £250 yearly. Despite all, the £100,000 needed by St. Thomas's is far from completion, £20,000 only having been so far received. Further particulars of the application of the Sussex scheme, which St. Thomas's, with the London and the Royal Free Hospitals, intends to put into practice as from October next, are therefore awaited with considerable interest.

### WIDER REPRESENTATION AT SHEFFIELD.

The Governors of the Royal Hospital, Sheffield, have decided to increase the number of the board of management from fifteen to twenty-five in order to give to the workers a larger share in the management. The recommendation of the hospital council was introduced by Mr. Philip K. Waise, the chairman of the governors, who said that hitherto the working classes of Sheffield had only one representative upon the board. All will agree that their valuable support entitles them to better representation and the amended rule makes this possible. The change is doubly opportune because it is due and because of the new scheme for collecting 1d. in the £ from the men in the various works in Sheffield. It should increase their interest in the hospital.

### SWANSEA'S LEAD.

Hospitals in all parts of the country have suffered severely from the industrial depression and the coal-mines stoppage, which have had their effect on the flow of contributions into the coffers from the workmen. When every penny of money is needed this is indeed a serious loss, and it may be helpful to hospital finance committees to know how Swansea is meeting the situation. The works governors of the hospital recommended the workmen to double their subscriptions when they resumed work, and it is stated that the spirit in which the recommendation has been received is a guarantee that within six or nine months the men will have made up all the leeway lost during their months of idleness.

